# High frequency of carpal tunnel syndrome and associated factors: A cross-sectional study in Peruvian workers from agro-export industry

**DOI:** 10.1097/MD.0000000000035927

**Published:** 2023-11-03

**Authors:** Rosalinda Tassara, Jorge Inolopú, Liliana Cruz-Ausejo, Kevin Jesús Mayma, Fernando Soncco-Llulluy, Jaime Rosales-Rimache

**Affiliations:** a Universidad Alas Peruanas, Lima, Peru; b Centro Nacional de Salud Ocupacional y Protección del Ambiente para la Salud, Instituto Nacional de Salud, Lima, Peru; c Universidad César Vallejo, Trujillo, Peru; d Vicerectorado de Investigación, Universidad Privada Norbert Wiener, Lima, Peru; e Escuela Profesional de Tecnología Médica, Universidad Continental, Lima, Peru.

**Keywords:** agricultural workers, agro-export workers, carpal tunnel syndrome, farmers, Phalen test, Tinel test

## Abstract

Carpal tunnel syndrome (CTS) is a peripheral mononeuropathy caused by compression of the median nerve at the wrist and has been reported in workers who perform repetitive movements that involve actions of sustained grasping of vibrating objects. We carried out a cross-sectional analytical study in March 2018 to identify the factors associated with the CTS among workers of agro-export companies in Ica-Peru. CTS confirmation in our study was based on having at least 1 positive screening test (Tinel or Phalen) and a presumptive result of CTS by the Kamath and Stothard Questionnaire. We carried out 4 Poisson regression models to evaluate the factors associated with CTS based on epidemiological and statistical criteria. We enrolled 112 agro-export workers in production (42.0%), packing (35.7%), and administration (22.3%) working areas. The CTS frequency in Peruvian agro-export workers in production, packing, and administrative working area were 78.7%, 45.0%, and 28.0%, respectively. The bivariate analysis found a relationship between the CTS with age, female sex, sports practice, job seniority in the working area (year), and repetitive wrist movements (hours per day). In the multivariate analysis, only job seniority in the working area (year) and repetitive wrist movements maintained their association with CTS. Occupational factors are significantly associated with a high frequency of CTS, such as job seniority in the working area (year) and repetitive wrist movements in agro-export workers. Surveillance programs should be held to prevent, reduce, and monitor workers’ health status.

## 1. Introduction

Carpal tunnel syndrome (CTS) is a peripheral mononeuropathy caused by a decrease in the diameter of the carpal tunnel lumen or inflammation of its structures that compresses the median nerve against the transverse ligament of the wrist.^[1,2]^ Such circumstances may arise congenitally or traumatically; however, idiopathic diagnosis is the most common.^[3,4]^ CTS is associated with medical disorders such as rheumatoid arthritis, endocrinological conditions, gout, hypertension, diabetes, and obesity, among others,^[5]^ non-medical disorders such as female gender, menopause, and pregnancy,^[6]^ and occupational activities.^[7]^ In this regard, CTS has been reported in workers who perform repetitive movements of the hands and wrists,^[8]^ actions involving sustained grasping of vibrating objects,^[9]^ repetitive wrist flexion-extension movements, and mechanical stress on the palmar base of the hand.^[10]^ Performing manual tasks that involve repeated flexion, extension, or ulnar deviation of the wrist, vibrations, and mechanical stress on the palmar base increases the risk of CTS.^[11–13]^

In this sense, CTS can manifest in both white-collar and blue-collar workers engaged in manual activities,^[14]^ encompassing cooks, cashiers, hairdressers, weavers, musicians, seamstresses, and other workers.^[15]^

The labor aspects associated with the development of STC described above lead us to consider farmers and the activities they carry out as potential triggers for STC. This is especially relevant in countries like Peru, a South American country that has experienced a significant increase in agricultural exports based on the development of agro-export companies in coastal regions. Agricultural exports are vital for the economies of developing regions in Peru and predominantly rely on manual activities for harvesting and packaging products due to the limited availability of advanced technology.^[16]^ Peruvian farmers involved in such agro-export companies, called agro-export workers, are typically day laborers or piece-workers who depend on their productivity for income.^[17]^ These informal working conditions, with limited social benefits, often exclude many Peruvian agro-export workers from undergoing regular medical evaluations within occupational health programs. Therefore, given the absence of reports in this occupational group in Peru, our study aims to assess the frequency of CTS and identify associated risk factors among Peruvian agro-export workers in a town on the southern coast of Peru. Our study is relevant due to the growing number of workers involved in agricultural activities and their limited access to the occupational health surveillance system.

## 2. Methodology

### 2.1. Study design and participants

In March 2018, we conducted a cross-sectional study aimed at evaluating Peruvian workers from 3 areas: the production, packing, and administrative areas of a company dedicated to the agro-export of fruits such as grapes, mangoes, blueberries, avocados, pomegranates, and others. The company was located in the district of Salas, in the department of Ica, which is 300 km south of the capital city of Lima, Peru. During the working day, after obtaining prior informed consent, we conducted 2 screening tests: the Tinel and Phalen test, and administered the Kamath and Stothard Clinical Questionnaire (KSQ). In this research, we considered a presumptive diagnosis for CTS when a positive response was elicited in the Tinel and/or Phalen test and was confirmed with the KSQ, contributing to increased specificity. We included male and female workers with a minimum job seniority in the working area of 6 continuous months, and we excluded individuals who were pregnant or had conditions such as hypothyroidism, arthritis, osteoarthritis, or upper limb injuries.

### 2.2. Techniques and instruments

#### 2.2.1. Epidemiological survey.

The epidemiological survey included a self-report questionnaire consisting of 3 sections: demographic information, occupational information, and symptomatology related to CTS. We obtained demographic information such as age, sex, body mass index, smoking, sports practice, and health history (diabetes mellitus and heart disease). The occupational information section included details about the work area, job seniority in the working area (year), frequency of repetitive wrist movements (measured in hours per day), and use of vibrating tools. The symptomatology related to the CTS section encompassed difficulties in grasping objects, numbness, tingling, swelling in the fingers, pain in the hand or wrist, fine motor coordination, decreased tactile, thermal perception, and thenar atrophy. The epidemiological survey was evaluated by 3 experts who dichotomously evaluated (“yes” or “no”) the relevance of the questions as part of content validity process. Our study’s calculated Kuder-Richardson coefficient (KR-20) was 92.5%, indicating high consistency and internal reliability of the questions.

#### 2.2.2. Functional screening tests.

We performed the Tinel and Phalen tests as screening methods. Previous studies have determined that both tests have a sensitivity and specificity ranging between 62% to 85% and 90% to 93%, respectively.^[18]^ Tinel’s test consisted of applying blows with the fingers on the wrist’s annular ligament and exerting pressure.^[19]^ For the Tinel test, we assessed the presence of channel involvement by identifying a cramping sensation over the first, second, and third fingers. For the Phalen test, we instruct the worker to perform a palmar flexion movement of the wrist at 90° for 1 minute.^[20]^ A positive response to the Phalen and Tinel tests was determined when paresthesia was elicited in the segments innervated by the median nerve, along with the presence of characteristic pain caused by the reduction of the carpal tunnel space, resulting in paresthesia in the hand.

#### 2.2.3. Kamath and Stothard Questionnaire.

Considered a tool for nerve conduction tests, the KSQ has a sensitivity of 85% compared to electrophysiological evaluation and a positive predictive value of 90%.^[21]^ This test is used for the confirmatory diagnosis of CTS and has been validated by electrophysiologists and hand surgeons in a randomized, single-masked design. The questionnaire consists of 9 items, and a total score equal to or greater than 3.0 provides the presumptive diagnosis of CTS.

### 2.3. Statistical analysis

We use frequencies and measures of central tendency to represent demographic and employment characteristics. We compared the distribution of the categorical and numeric independent variables concerning the CTS using the chi-square test or the Mann-Whitney test for median comparison, respectively, considering a value of *P* < .05 as a significant difference. Identifying factors associated with CTS was evaluated using 4 Poisson models with robust variance in which we calculated the adjusted prevalence ratios, considering Akaike’s information criteria. We use Stata software version 17 (StataCorp, College Station, TX) y GraphPad Prism software.

### 2.4. Ethical approval

The study was approved by the Universidad Alas Peruanas Ethics Committee, with resolution RD N°089-2018-EPTM-FCS-UAP. We obtained permission from the agro-export company to evaluate the active workers on site. We obtained the informed consent of each participating worker after informing them of the objectives, use of instruments, benefits, risks, and confidentiality of the information.

## 3. Results

**Figure 1.**
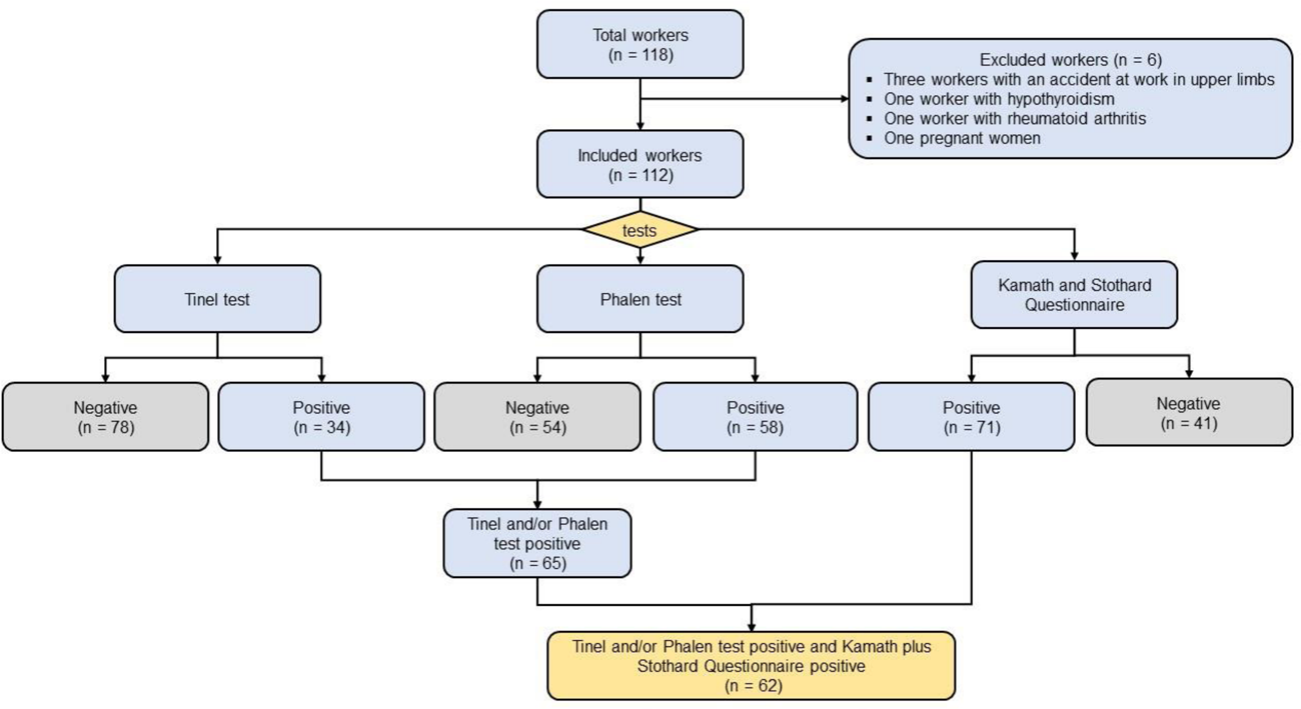
Participant enrollment flowchart.

The evaluated agro-export company had 118 Peruvian agro-export workers, of which 6 were excluded, leaving 112 workers in the study to whom the Tinel, Phalen, and KSQ tests were applied (Fig. 1). The enrolled workers had a median age of 32 (interquartile range = 26–42) years, were mainly male (55.4%), and were overweight primarily (51.8%) (Table 1). The report of smoking was reported in 12 (11.2%) workers. Participants who smoke reported smoking at a frequency of 1 cigarette per day. The practice of sports was declared by almost half of the workers (48.7%).

**Table 1:** Characteristics of Peruvian agro-export workers (n = 112).

	n (%)	95% CI
Age (yr)	32 (26–42)*	
Sex		
Male	62 (55.4)	45.9–64.4
Female	50 (44.6)	35.6–54.1
Cigarette smoking	12 (11.2)	6.4–18.9
Sports practice	54 (48.7)	39.4–58.0
Body mass index (BMI) (kg/m^2^)		
Normal weight (BMI between 25 and 29.9)	43 (38.4)	29.7–47.8
Overweight (BMI between 25 and 29.9)	58 (51.8)	42.4–61.0
Obesity (BMI ≥ 30)	11 (9.8)	5.5–17.0
Health history		
Heart disease	4 (3.6)	1.3–9.3
Mellitus diabetes	2 (1.8)	0.4–6.9
Work area		
Production	47 (42.0)	33.1–51.4
Packing	40 (35.7)	27.3–45.1
Administration	25 (22.3)	15.5–31.1
Job seniority in working area (years)	4 (2–5)*	
Repetitive wrist movements (hours per day)	6 (3–7)*	
Use of vibration tools	17 (15.2)	9.5–23.2

* p50 (ric): median (interquartile range).

Regarding working conditions, the workers reported belonging to 3 work areas: production (42.0%), packing (35.7%), and administration (22.3%), with a median job seniority in the working area of 4 years (interquartile range = 2–5). All workers reported working 8 hours daily and performing repetitive wrist movements with a median of 6 hours per day (interquartile range = 3–7), respectively. Likewise, some workers (15.2%) reported using instruments and equipment emitting vibrations for approximately 3 hours daily. The frequency of repetitive wrist movements determined by the median number of hours per day was significantly higher in the workers in the production area compared to the packing areas (*P* < .01), and the latter was significantly higher than the workers in the packing area and administration (*P* < .01).

**Figure 2.**
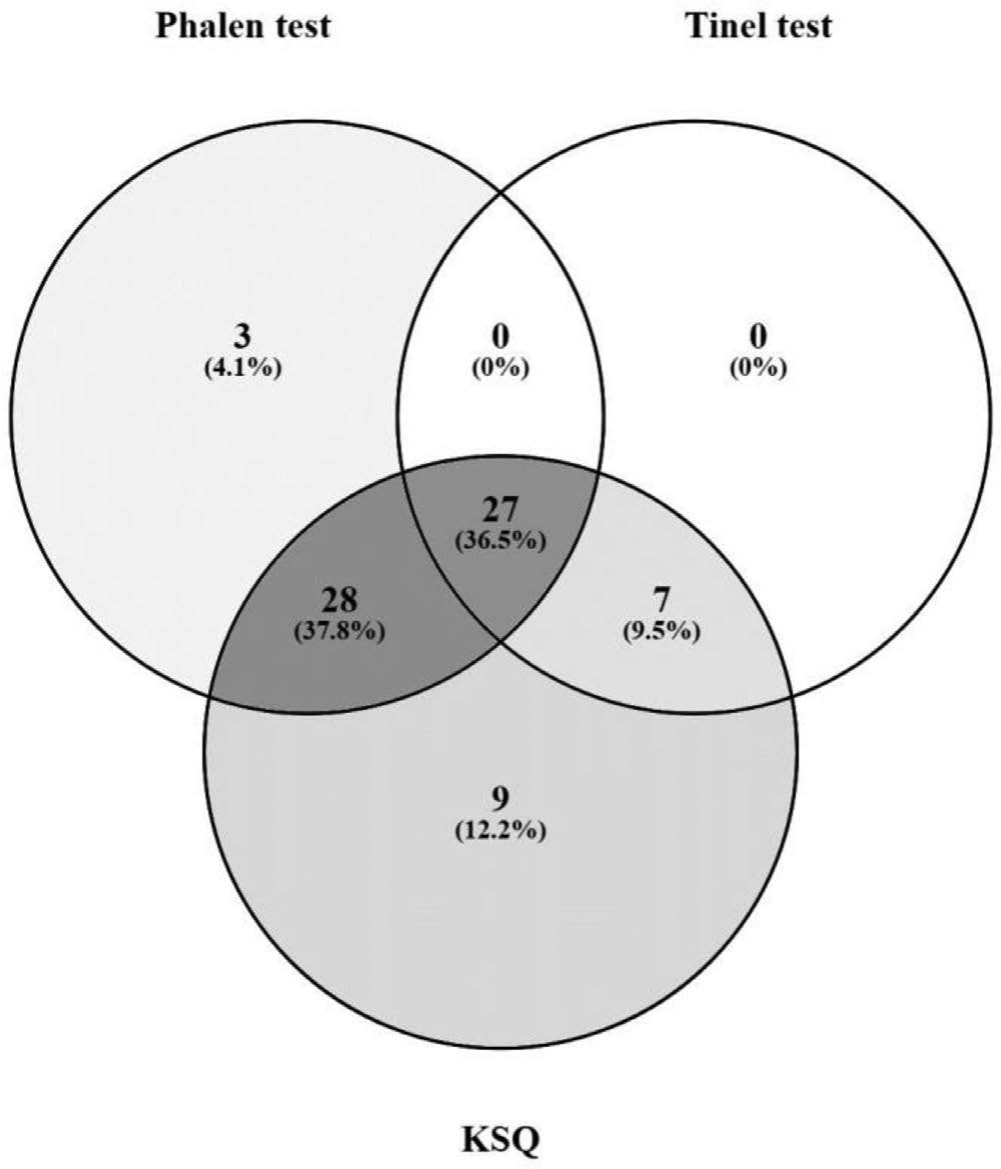
Distribution of positive results by the Tinel test, the Phalen test, and the Kamath and Stothard Questionnaire (KSQ) using a Venn diagram.

**Figure 3.**
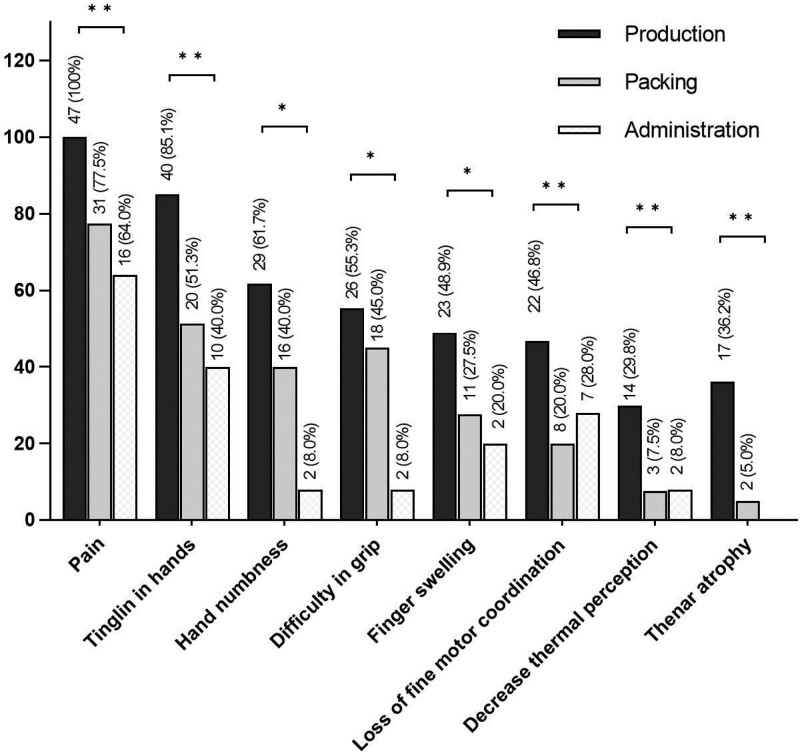
Frequency distribution of signs of CTS according to the work area by bar graph. CTS = Carpal tunnel syndrome.

In this regard, 62 (55.4%, 95% confidence interval (95% CI): 45.0–64.4) workers had confirmed CTS. The KSQ test identified 71 (63.4%) workers with CST, while the Tinel and Phalen tests identified 34 (30.4%) and 58 (51.8%) workers with CST, respectively (Fig. 2). In the production area, the CTS was present in 37 (78.7%, 95% CI: 64.2–88.4) workers; in the packing area, the CTS was present in 18 (45%, 95% CI: 29.9–61.1) workers, and in the administrative area the CTS was present in 7 (28%, 95% CI: 13.2–49.8) workers. On the other hand, the most frequent manifestations related to CTS were wrist pain (83.9%), tingling (63.1%), numbness in the hands (42.0%), and difficulty gripping (41.1%), mainly. The workers in the production area showed a significantly higher frequency in all the manifestations evaluated compared to other areas (*P* < .05), except in the difficulty of grasping (Fig. 3).

Regarding the bivariate analysis (Table 2), the CTS was related to a greater age (*P* = .021), to the female sex (*P* = .016), and the practice of sport (*P* < .001). Likewise, the CTS was related to belonging to the production area (*P* < .001), to a longer stay in the work area (*P* = .005), to a higher frequency of repetitive wrist movements (*P* < .001), and, paradoxically, to less use of vibration tools (*P* = .019). Finally, we performed 4 Poisson regression models with robust variance to determine CTS prevalence ratios in agro-export workers, prioritizing factors based on epidemiological criteria, and the assessment was completed with Akaike’s information criteria (AIC) (Table 3). Model 1 contains the variables age (*P* = .98), sex (*P* = .08), and sports practice (*P* = .02), model 2 includes job seniority in working area (year) (*P* = .004), model 3 includes the frequency of repetitive wrist movements (*P* = .030) and model 4 includes the use of vibration tools (*P* = .201). The variability of the AIC was greater for model 3, in addition to presenting the highest goodness-of-fit coefficient (0.9999). The Poisson distribution was confirmed using the Pearson goodness-of-fit coefficient (*P* > .05) for the 4 models.

**Table 2:** Factors related to carpal tunnel syndrome in Peruvian agro-export workers (n = 112).

Characteristics	Carpal tunnel syndrome		*P* value
	Not confirmed (n = 50)	Confirmed (n = 62)	
Age (yr)	29 (25–36)*	35 (27–46)*	.021†
Sex			
Male	34 (68.0)	28 (45.2)	.016‡
Female	16 (32.0)	34 (54.8)	
Smoking	8 (17.0)	4 (6.7)	.419‡
Sports practice	32 (64.0)	22 (36.1)	<.001‡
Work area			
Production	10 (20.0)	37 (59.7)	<.001‡
Packing	22 (44.0)	18 (29.0)	
Administration	18 (36.0)	7 (11.3)	
Job seniority in working area (yr)	2 (2–4)*	4 (1–7)*	.005†
Repetitive wrist movements (hours per day)	6 (3–6)	7 (6–7)	<.001†
Use of vibration tools	12 (24.0)	5 (8.6)	.019‡

* (p50 (IQR)).

† Mann–Whitney nonparametric test.

‡ Pearson’s chi-squared test.

**Table 3:** Factors independently associated with carpal tunnel syndrome in Peruvian agro-exportation workers by 5 Poisson regression analysis models (n = 112).

Carpal tunnel syndrome	cPR*	95% CI	*P* value	Model 1			Model 2			Model 3			Model 4		
				aPR†	95% CI	*P* value	aPR†	95% CI	*P* value	aPR†	95% CI	*P* value	aPR†	95% CI	*P* value
Age (yr)	1	(0.99–1.02)	.391	1	(0.99–1.01)	.98	1	(0.98–1.01)	.426	1	(0.99–1.01)	.978	0.99	(0.99–1.01)	.93
Sex															
Male	Ref.			Ref.		Ref.	Ref.		Ref.	Ref.			Ref.		
Female	1.5	(1.08–2.10)	**.02**	1.36	(0.96–1.91)	.08	1.33	(0.93–1.88)	.113	1.15	(0.83–1.59)	.411	1.13	(0.82–1.56)	.457
Sport practice															
No	Ref.			Ref.		Ref.	Ref.		Ref.	Ref.			Ref.		
Yes	0.59	(0.41–0.86)	**<.01**	0.63	(0.43–0.93)	**.02**	0.68	(0.46–0.01)	.059	0.86	(0.58–1.25)	.424	0.86	(0.59–1.26)	.440
Job seniority in working area (year)	1.09	(1.05–1.13)	**<.01**				1.08	(1.03–1.134)	**.004**	1.06	(1.01–1.12)	**.016**	1.06	(1.01–1.11)	**.015**
Repetitive wrist movements (hours per day)	1.22	(1.06–1.40)	**<.01**							1.16	(1.01–1.34)	**.038**	1.15	(1.00–1.31)	.052
Use of vibration tools															
No	ref.														
Yes	0.49	(0.23–1.05)	.065										0.70	(0.33–1.50)	.355
Akaike’s Information Criteria (AIC)‡				197.75			197.11			**195.66**			197.07		
Pearson’s goodness of fit test				0.9989			0.9994			**0.9998**			0.9997		

*P*-values in bold are significant.

aPR = Adjusted prevalence ratio, CI = Confidence interval, cPR = Crude prevalence ratio.

* Crude prevalence ratio by Poisson regress.

† Adjusted prevalence ratio by Poisson regress.

‡ Consider that the initial AIC was 199.33.

## 4. Discussion

The frequency of CTS in the evaluated Peruvian agro-export workers was 55.4%. Among the different areas of the agro-export company, workers in the production area exhibited the highest CTS frequency (78.7%), along with the highest symptomatology compared to workers in the packing and administrative areas. Likewise, the frequency of CST in workers in the packing area was found to be 45%. The prevalence of CTS in the general Peruvian population has yet to be reported, and reporting on CTS in agricultural workers in the world is limited. There is evidence of the presence of CTS in Italian vineyard farmers, who had different electrophysiological characteristics compared to workers, specifically greater involvement of the fibers of the thenar motor branch.^[22,23]^ Likewise, evidence suggests that tea harvesting might be an occupational risk factor for work-related CTS development.^[24]^ The observed high prevalence and gradient frequency of CTS in our study, along with its associated clinical manifestations such as hand pain, numbness, tingling, and difficulty in grasping, among workers in the production, packing, and administration areas, indicate the significant impact of labor-related factors on the development of CTS strongly suggesting its relationship with work activities in the work area.

In this regard, the workers in the production area of the agro-export company evaluated were involved in harvesting activities for various fruits, including mangoes, pomegranates, grapes, blueberries, and avocados. These tasks were carried out using harvesting shears equipped with a resistance mechanism that facilitated the opening of the cutting blades. This task implies the performance of pincer movements between the first and the rest of the fingers with the dominant hand, exerted by the contraction of the muscles mainly in the thenar zone in synergy with the extensor muscles of the fingers. On the other hand, the workers in the packing area engage in rotational trunk movements, accompanied by various movements of the upper limbs. These movements include pronation-supination (rotation of the forearm), flexion-extension of the elbows, and flexion-extension of the wrists and are developed during the selection of the products that pass-through conveyor belts. These activities are accompanied by full hand press movements to hold large fruits (mangoes, pomegranates, etc.) or distal press to manipulate small fruits (grapes and blueberries, etc.); both movements require dexterity, coordination, and fine motor skills.

In the bivariate analysis, our results showed that female sex and older age were related to CTS. However, this relationship is lost in models 1 to 4 in the multivariate analysis. Smoking habits were not found to be related to CTS in our study; therefore, they were not included in the multivariate analysis. In contrast, the practice of sport showed a relationship with the presence of CTS in the bivariate analysis and model 1 of the multivariate analysis. However, this relationship is lost in models 2 to 4. The population that reported practicing sport referred to soccer with greater frequency, which is not a factor associated with CTS since it does not necessarily imply a significant load on the wrist, hand, or upper limbs. Among the models evaluated, model 3 was selected, which shows a light association between job seniority years in the working area (adjusted OR = 1.06, 95% CI: 1.01–1.12) and repetitive wrist movements based on hours per day (adjusted OR = 1.16, 95% CI: 1.01–1.34).

Our results showed that the higher frequency of repetitive wrist movements was associated with CTS in model 3, according to what has been reported in various studies.^[11–13]^ In this regard, a study on Italian workers from meat processing industries showed that prolonged wrist vending was related to an increased CTS diagnosis (adjusted OR = 2.561, 95% CI: 1.10–5.96).^[25]^ Likewise, a study reported that the prevalence of CTS among US dairy parlor workers was 16.6% and 3.6% among non-parlor workers, with a statistically significant difference (adjusted OR = 5.3, 95% CI: 1.1–25.5).^[26]^ Another study of healthcare and manufacturing workers showed that the increase in manual activity was related to the increase in the prevalence of CTS, which increased in the presence of obesity.^[27]^ Finally, a study on male French Farmers and agricultural workers showed that 44 workers (5.6%, 95% CI: 4.0–7.7) reported suffering from unilateral/bilateral CTS during the last 12 months. It was related to physical wrist exposure performing more than 2 actions per minute for > 4 hours per day.^[28]^ Our study demonstrated that job seniority was significantly associated with CTS in models 2 to 4 of the multivariate analysis, in line with findings from a study that revealed a relation between CTS and increased job seniority (3–5 years) among visual display terminal workers in Taiwan.^[29]^

Finally, this study has some limitations. Firstly, due to its cross-sectional study design, we can not extrapolate or generalize the results to populations with similar characteristics. We must consider that the results obtained from our study have strict applicability and are limited to the agro-export company that was evaluated. Therefore, we recommend interpreting these results with caution. Secondly, some workers with symptomatic conditions may have chosen to participate or not in the study, either out of interest or out of fear of acknowledging an injury that could be exposed. Therefore, we believe that a selection bias might have been introduced. Thirdly, it is essential to acknowledge that the development of CTS may be influenced by other factors that were not specifically evaluated in our study. Psychosocial factors like work stress, Burnout syndrome, and anxiety states have been associated with an increased risk of CTS.^[30]^

While the findings may suggest common patterns among companies with similar activities, it is necessary to exercise caution when generalizing the results to other contexts. However, reports on labor conditions in the Department of Ica show that day labor and piecework activities are predominant.^[31]^ The expansion of agro-export activity in the department of Ica, Peru, demands a growing workforce that operates in unfavorable working conditions, which can predispose individuals to the development of CTS and other occupational disorders. Indeed, the 2017 Peruvian Household Survey revealed that approximately 8 million Peruvians, representing individuals from various poverty levels, relied on the agricultural sector as a livelihood.^[32]^ Given this scenario, it becomes crucial to promptly identify and address individual and occupational factors closely linked to CTS and related disorders among agro-export workers. Implementing effective occupational health surveillance measures is crucial for early detection and intervention.

## 5. Conclusion

The frequency of CTS in the workers of the production area was high, and it is associated with job seniority and the frequency of repetitive wrist movements. These factors, specific to work, must be considered by the Occupational Health and Safety Services for the evaluation, design of programs, surveillance, and monitoring of workers’ health. We recommend ergonomically characterizing the workplace, and working conditions (number of workdays, work modality: piecework or per day, rotating shifts, etc.) and designing a study with sufficient validity to establish whether the working conditions in Peruvian agro-export workers contribute to the development of CTS and other neuropathies.

## Acknowledgments

The Universidad Científica del Sur support in paying the article processing charge of this work.

## Author contributions

**Conceptualization:** Tassara Rosalinda, Rosales-Rimache Jaime.

**Formal analysis:** Tassara Rosalinda, Inolopú Jorge, Cruz-Ausejo Liliana, Rosales-Rimache Jaime.

**Investigation:** Tassara Rosalinda, Inolopú Jorge, Mayma Kevin Jesús, Soncco Fernando.

**Methodology:** Tassara Rosalinda, Cruz-Ausejo Liliana, Mayma Kevin Jesús, Soncco Fernando, Rosales-Rimache Jaime.

**Project administration:** Tassara Rosalinda.

**Supervision:** Rosales-Rimache Jaime.

**Writing – original draft:** Tassara Rosalinda, Inolopú Jorge, Cruz-Ausejo Liliana, Mayma Kevin Jesús, Soncco Fernando.

**Writing – review & editing:** Cruz-Ausejo Liliana, Rosales-Rimache Jaime.
